# Comparación entre mediciones e índices antropométricos para evaluar la obesidad general y la abdominal, Colombia ENSIN 2015

**DOI:** 10.7705/biomedica.7011

**Published:** 2023-12-29

**Authors:** Ana Yibby Forero, Luis Carlos Forero

**Affiliations:** 1 Grupo de Nutrición, Dirección de Investigación en Salud Pública, Instituto Nacional de Salud, Bogotá, D.C., Colombia Instituto Nacional de Salud Grupo de Nutrición Dirección de Investigación en Salud Pública Instituto Nacional de Salud Bogotá, D.C. Colombia

**Keywords:** antropometría, índice de masa corporal, obesidad abdominal, encuestas nutricionales, adulto, salud, Colombia, anthropometry, body mass index, obesity, abdominal, nutrition surveys, adult, health, Colombia

## Abstract

**Introducción.:**

El exceso de peso representa un problema en la población adulta, el cual aumentó en los últimos años y se asocia con enfermedades no transmisibles.

**Objetivos.:**

Comparar las mediciones con los índices antropométricos y evaluar su relación con variables individuales y sociodemográficas para generar información sobre el uso de las principales medidas en la evaluación de la obesidad general y la abdominal como indicadores de riesgo cardiovascular.

**Materiales y métodos.:**

Se hace un análisis secundario de la encuesta ENSIN para Colombia, con datos recolectados entre el 2015 y el 2016. Como marco muestral, se utilizó el Censo de Población y Vivienda del 2005 del Departamento Administrativo Nacional de Estadística (DANE), y la muestra incluyó 44.202 hogares y 151.343 personas de 0 a 64 años; para este análisis, se seleccionaron 70.315 registros de población adulta. Se calcularon proporciones, intervalos de confianza, medidas de tendencia central y dispersión.

**Resultados.:**

Las medias del índice de masa corporal (IMC) y del índice cintura-estatura fueron más grandes que el punto de corte en ambos sexos, mientras que las medias de la circunferencia de la cintura estuvieron por debajo del corte para los hombres y por encima de aquel para las mujeres (p<0,05). La prevalencia de obesidad por IMC fue de 17,5 %, mientras que la obesidad abdominal, según la circunferencia de la cintura, fue de 50,2 %, y según el índice cintura-estatura, de 62,6 %.

**Conclusiones.:**

Independientemente del indicador utilizado, la obesidad abdominal es cerca de tres veces más frecuente que la obesidad general por IMC; con el índice cintura- estatura se identifican más personas, en especial hombres, con obesidad abdominal en comparación con la circunferencia de la cintura.

## Abstract (en)

**Introduction.:**

Excess weight represents a problem in the adult population, has increased in recent years and is associated with noncommunicable diseases.

**Objectives.:**

To make comparisons between anthropometric measurements and indices and to evaluate their relationship with individual and sociodemographic variables to generate information on the use of the main measurements in the evaluation of general and abdominal obesity as indicators of cardiovascular risk.

**Materials and methods:**

. Secondary analysis of the ENSIN survey for Colombia with data collected between 2015 and 2016, the 2005 Population and Housing Census of the *Departamento Administrativo Nacional de Estadística* (DANE) was used as a sampling frame. The sample was 44,202 households with 151,343 people aged 0 to 64 years; 70,315 records of adult population were selected for this analysis. Proportions, confidence intervals, measures of central tendency and dispersion were calculated.

**Results.:**

The means of body mass index (BMI) and waist-height index were higher than the cutoff point in both sexes, while the means of waist circumference were below the cutoff point for men and higher for women (p<0.05). The prevalence of obesity by BMI was 17.5%, while abdominal obesity by waist circumference and waist-height index of 50.2% and 62.6% respectively.

**Conclusions.:**

Abdominal obesity regardless of the indicator used, is about 3 times higher than general obesity by BMI and waist-height index identifies more people, especially men, with abdominal obesity compared to waist circumference.

El exceso de peso, que incluye el sobrepeso y la obesidad, constituye un problema actual de la población adulta y en los últimos años ha presentado un continuo aumento ^1^. En el mundo se ha triplicado entre 1975 y 2016, afectando a más de 1,9 billones de adultos ^2^, lo que corresponde a más de la mitad (52 %) de la población ^3^.

En el contexto colombiano, según la Encuesta Nacional de la Situación Nutricional (ENSIN), el exceso de peso en adultos se incrementó del 51,2 % en el 2010 al 56,5 % en el 2015 y, la obesidad, del 16,5 % al 18,7 %, respectivamente ^4^. Además, la obesidad se asocia con enfermedades no transmisibles como la hipertensión arterial sistémica, las dislipidemias, la diabetes mellitus de tipo 2 y las cardiovasculares, consideradas las principales causas de muerte ^5^.

Para clasificar la obesidad general y abdominal, se han utilizado medidas antropométricas directas como el peso, la estatura y la circunferencia de cintura, e índices antropométricos como el índice de masa corporal (IMC) y el índice cintura-estatura. Estas mediciones e índices pueden tener limitaciones que pueden inducir a la clasificación errónea de los individuos por no tener en cuenta sus características de composición corporal y altura; por ejemplo, se puede diagnosticar sobrepeso u obesidad en personas con una gran masa muscular ^6^.

El IMC, validado por la Organización Mundial de la Salud (OMS), es generalmente el utilizado para determinar el estado nutricional, porque permite clasificar el sobrepeso, la obesidad y su magnitud en la población, en diferentes contextos ^7^; sin embargo, a pesar de que evalúa tanto la normalidad como la anormalidad del peso corporal, dicho índice no discrimina entre el componente graso y el magro ^8^. Además, algunos estudios han demostrado aumento de la frecuencia del síndrome metabólico cuando se incrementa este índice; no obstante, no se considera una medida relacionada con la distribución o la localización del exceso de grasa ^7^.

Entre las mediciones de la distribución de adiposidad central o de obesidad abdominal, la más utilizada es la circunferencia de cintura, que se considera un factor predictor de riesgo cardiovascular porque indica acumulación de grasa abdominal ^9^ y, de manera indirecta, de obesidad localizada; además, es una medida segura, rápida y de fácil interpretación ^10^. Algunos estudios realizados con tomografía axial computarizada y antropometría, demostraron una sólida asociación entre la circunferencia de cintura y la grasa abdominal ^11^.

Recientemente, el índice cintura-estatura fue descrito como un elemento predictor del riesgo cardiovascular por considerarse un buen estimador de la grasa visceral, cuyo uso se justifica pues permite una medida ajustada de la circunferencia de cintura en función de la estatura; esto resulta en una estimación más acorde con las dimensiones del individuo, siendo este índice más específico que el IMC ^12^. Además, el índice cintura-estatura contribuye a predecir la morbimortalidad en diversas poblaciones, sin importar el sexo ni la edad ^13^.

Las anteriores medidas e índices cobran importancia en la determinación del exceso de peso y la acumulación de grasa corporal. Sin embargo, la limitada evidencia sobre la capacidad del IMC y el índice cintura-estatura en relación con la circunferencia de cintura para predecir riesgos en salud, como la enfermedad cardiovascular, hace necesario comparar estas medidas e índices en Colombia. De esta forma, en este estudio se planteó comparar las mediciones con los índices antropométricos, y evaluar su relación con variables individuales y sociodemográficas; todo esto, con el fin de generar conocimiento e información sobre el uso de las principales medidas en la evaluación de la obesidad general y la abdominal como indicadores de riesgo cardiovascular.

## Materiales y métodos

Se hizo un análisis secundario de la información recolectada en la Encuesta Nacional de la Situación Nutricional en Colombia ENSIN 2015, la cual es una encuesta poblacional, de corte transversal, en la que se utilizó un muestreo probabilístico y polietápico por conglomerados, con cobertura y representatividad nacional, en las áreas urbana y rural, para seis regiones y para las mediciones antropométricas desagregadas en 14 subregiones y 32 departamentos del país. Se utilizó la base de datos anonimizada del componente de indicadores antropométricos para estimar el estado nutricional ^4^.

El universo de la encuesta estuvo constituido por la población civil no institucional residente habitual de los hogares de todo el territorio nacional; se utilizó como marco muestral el Censo de Población y Vivienda del 2005 del Departamento Administrativo Nacional de Estadística (DANE). La encuesta efectiva se llevó a cabo en una muestra de 44.202 hogares distribuidos en 4.739 segmentos de 177 estratos, en 295 municipios de todos los departamentos del país y Bogotá; se encontraron 151.343 personas de 0 a 64 años, de las cuales se presentaron resultados de 74.922 personas entre los 18 y los 64 años ^4^.

La recolección de la información del operativo de campo se llevó a cabo entre el 2015 y el 2016. Para la ENSIN 2015, se diseñó y ejecutó un sistema de captura como herramienta de apoyo para la recolección, con una interfaz que incluía los aspectos de control más relevantes para la correcta aplicación de la metodología ^4^.

Para este estudio, se depuró la base de datos, teniendo en cuenta que todos los individuos tuvieran las medidas antropométricas requeridas y, con base en la distribución de los datos, se suprimieron las observaciones poco plausibles o extremas; en este caso, los registros inferiores y superiores a los percentiles 1 y 99, respectivamente. De esta forma, la muestra final para el análisis fue de 70.315 registros.

Las medidas antropométricas utilizadas fueron el peso en kg, y la estatura y la circunferencia de cintura en cm, a partir de las cuales se calculó el IMC, siguiendo la fórmula de Quetelet que lo define como la relación entre el peso y la estatura , y el índice cintura-estatura como el cociente entre la circunferencia de la cintura y la estatura en centímetros. El peso fue tomado con una balanza electrónica (SECA-874™); la estatura, con un tallímetro de referencia (Shortboard™), y la circunferencia de la cintura, con una cinta métrica de material metálico (Rosscraft™). Además, se consideraron otras covariables para hacer desagregaciones y comparaciones, como sexo, edad, etnia, área de residencia, región e índice de riqueza.

Los puntos de corte utilizados para clasificar la obesidad fueron los siguientes: en el caso de la obesidad general, un IMC ≥30 kg/m^2^, según lo establecido por la OMS; para la obesidad abdominal, la circunferencia de la cintura, según lo establecido por el Consenso Colombiano de Síndrome Metabólico, en mujeres, ≥80 cm y, en hombres, ≥90 cm ^14^; y, también, para la obesidad abdominal, el índice cintura-estatura ≥0,5, que corresponde al límite referido por algunos autores ^7^^,^^8^^,^^12^. Teniendo en cuenta lo anterior, se crearon variables categorizadas con respuesta dicotómica, de quienes presentaban obesidad y de quienes no la presentaban.

Para analizar los datos, se utilizó el programa Stata™, versión 12, y el comando *svy* para muestras complejas de datos provenientes de encuestas, a partir del cual se estimaron proporciones e intervalos de confianza del 95 %, así como medidas de tendencia central y dispersión ajustadas por el diseño de la encuesta. Para las comparaciones estadísticas, en el caso de las variables categóricas, se empleó la prueba estadística de ji al cuadrado y, para evaluar la diferencia entre los grupos de las variables continuas, se ejecutó el comando *lincom* en Stata, que calcula la t de Student y el valor de p de los coeficientes después de la estimación para encuestas (*svy*).

La ENSIN 2015 cumplió las consideraciones éticas vigentes en el país (Declaración de Helsinki y Resolución 8430 de 1993 del Ministerio de Salud), incluyendo el consentimiento informado de los participantes, y fue avalada por el Comité de Ética del Instituto Nacional de Salud según el Acta N°2-2015 del 16 de febrero de 2015. De acuerdo con el Artículo 11 de la Resolución 8430 de 1993, se estableció que este estudio no representa ningún riesgo, ya que es un análisis a profundidad a partir de la base de datos de una encuesta poblacional anonimizada, dispuesta y de acceso público en el Repositorio Institucional Digital del Ministerio de Salud y Protección Social y, también, se solicitó siguiendo el procedimiento indicado para su uso ^15^.

## Resultados

La mayor proporción de la población analizada fue de adultos jóvenes entre 18 y 29 años (32,6 %), el promedio de edad fue de 38,1 años, la proporción de mujeres fue 53,3 %, el 88,0 % se autorreconoció sin ninguna pertenencia étnica y el 30,3 % pertenecía al cuartil de riqueza más bajo. En cuanto al área de residencia, el 78,0 % de los hogares eran urbanos. Según la distribución por regiones del país, el 24,4 % de la población encuestada habitaba en la Central, el 21,5 % en la Atlántica, el 17,4 % en la Pacífica, el 17,3 % en la Oriental, el 17,1 % en Bogotá y el 2,3 % en la Orinoquía y Amazonía (cuadro 1).

**Cuadro 1:** Edad y datos antropométricos de la población según el sexo, Colombia 2015

Característica		Población total		Hombres		Mujeres		p^**^
Individuos (n)^*^		70.315		31.672		38.643		
Individuos (n) expandido		22’929.096		10’716.728		12’212.369		
		x̅ **- %** ^+^	IC_95_%^++^	x̅ **- %** ^+^	IC_95_%^++^	x̅ **- %** ^+^	IC_95_%^+ +^	
Edad (años)		38,1	(37,9 - 38,3)	37,6	(37,3 - 37,8)	38,5	(38,3 - 38,8)	0,00
Edad [años agrupada (%)]								0,00
	18 - 29	32,6	(31,9 - 33,3)	34,8	(33,8 - 35,8)	30,7	(29,9 - 31,5)	
	30 - 39	23,2	(22,6 - 23,8)	22,7	(21,8 - 23,6)	23,7	(23,0 - 24,4)	
	40 - 49	20,3	(19,5 - 21,1)	19,5	(18,3 - 20,7)	21,0	(20,2 - 21,7)	
	50 - 59	17,6	(17,1 - 18,2)	16,9	(16,2 - 17,6)	18,3	(17,6 - 19)	
	60 - 64	6,3	(5,9 - 6,6)	6,1	(5,6 - 6,6)	6,4	(6 ,0- 6,9)	
Peso (kg)		68,2	(68 - 68,4)	71,9	(71,6 - 72,2)	53,3	(52,7 - 53,8)	0,00
Estatura (cm)		161,6	(161,4 - 161,7)	167,8	(167,6 - 168)	156,1	(155,9 - 156,2)	0,00
IMC (kg/m2)		26,1	(26,1 - 26,2)	25,5	(25,4 - 25,6)	26,7	(26,6 - 26,8)	0,00
CC cm		85,1	(84,9 - 85,3)	86,6	(86,3 - 86,8)	83,8	(83,6 - 84,1)	0,00
ICE		0,528	(0,526 - 0,529)	0,516	(0,515 - 0,518)	0,538	(0,536 - 0,54)	0,00

IMC: índice de masa corporal circunferencia de cintura; CC: circunferencia de cintura; ICE: índice cintura-estatura

* Se incluyeron solo personas residentes habituales.

^**^ Prueba de ji al cuadrado (*x* ^2^) para las comparaciones de las proporciones en variables categóricas y estadística t para las continuas

+ Las variables categóricas se presentan en proporción (%) y en media para las continuas

++ Intervalo de confianza del 95 %

En relación con las medidas antropométricas, el peso y la estatura fueron significativamente mayores en los hombres (p=0,00); las medias del IMC fueron superiores al punto de corte en ambos sexos y levemente mayor en las mujeres; las medias de la circunferencia de la cintura estuvieron por debajo del punto de corte en los hombres y, por encima, en las mujeres (cuadro 1). La media del índice cintura-estatura presentó valores superiores al punto de corte, tanto en el sexo masculino como en el femenino; sin embargo, fueron significativamente mayores en las mujeres.

Analizando las medidas e índices antropométricos según la presencia de obesidad (cuadro 2), se encontró que la mayoría de las medias son superiores para la clasificación de obesidad general por IMC, en comparación con las encontradas utilizando la circunferencia de la cintura y el índice cintura-estatura, excepto para la estatura.

**Cuadro 2:** Medias de variables continuas, según obesidad general o la abdominal, Colombia 2015

Variable	^*^	Obesidad general		Obesidad abdominal		Obesidad abdominal	
		IMC		CC		ICE	
		Sí	No	Sí	No	Sí	No
Edad (años)	x̅	42,3	37,2	42,4	33,7	41,9	31,6
	**IC**_**95%**_	(41,9 - 42,7)	(37 - 37,4)	(42,2 - 42,7)	(33,5 - 33,9)	(41,7 - 42,2)	(31,4 - 31,9)
Peso (kg)	x̅	83,7	64,9	74,8	61,6	72,8	60,6
	**IC**_**95%**_	(83,4 - 84)	(64,8 - 65,1)	(74,5 - 75)	(61,4 - 61,8)	(72,5 - 73)	(60,4 - 60,8)
Estatura (cm)	x̅	159,3	162	160,4	162,8	160,2	163,8
	**IC**_**95%**_	(159,1 - 159,6)	(161,9 - 162,2)	(160,2 - 160,6)	(162,6 - 163)	(160,1 - 160,4)	(163,6 - 164)
IMC (kg/m2)	x̅	32,9	24,7	29	23,2	28,3	22,5
	**IC**_**95%**_	32,9 - 33)	(24,6 - 24,7)	(29 - 29,1)	(23,2 - 23,3)	(28,2 - 28,4)	(22,5 - 22,6)
CC (cm)	x̅	99,2	82,1	93,3	76,8	91,4	74,6
	**IC**_**95%**_	(98,9 - 99,5)	(81,9 - 82,3)	(93,1 - 93,5)	(76,7 - 77)	(91,2 - 91,6)	(74,5 - 74,8)
ICE	x̅	0,624	0,508	0,583	0,473	0,571	0,456
	**IC**_**95%**_	(0,622 - 0,626)	(0,506 - 0,509)	(0,581 - 0,584)	(0,472 - 0,474)	(0,57 - 0,572)	(0,455 - 0,457)

IMC: índice de masa corporal circunferencia de cintura; CC: circunferencia de cintura; ICE: índice cintura-estatura

^*^ Se presentan la media (x̅) y el intervalo de confianza del 95 % (IC_95%_).

Todas las diferencias entre los que tienen o no obesidad fueron significativas (estadística *t* para comparación de coeficientes después de la estimación de encuestas *svy*).

Independientemente del índice de clasificación de la obesidad utilizado, al comparar los datos, se observó que las medias (excepto la de la estatura) eran mayores en la población con obesidad; se encontró una diferencia de 10 a 20 kg en el peso, de 15 a 20 cm en la circunferencia de la cintura y de 5 a 8 puntos en el IMC. Todas estas diferencias fueron estadísticamente significativas (p=0,000).

La prevalencia de la obesidad según el IMC fue del 17,5 %, mientras que la de la obesidad abdominal según la circunferencia de la cintura fue del 50,2% y, según el índice cintura-estatura, del 62,6 %. De esta forma, el índice cintura-estatura y la circunferencia de la cintura captan más población que el IMC, con una diferencia estadísticamente significativa (p=0,000) (cuadro 3).

**Cuadro 3:** Prevalencia de obesidad en adultos, según variables sociodemográficas, Colombia 2015

Variable		n+	Obesidad general IMC		P^*^	Obesidad abdominal CC		P^*^	Obesidad abdomina ICE		P^*^
			%	IC_95%_		%	IC_95%_		%	IC_95%_	
Total nacional		70.315	17,5	(17,0 - 18,1)		50,2	(49,3 - 51,0)		62,6	(61,7 - 63,4)	
Sexo					0,000			0,000			0,000
	Masculino	31.672	12,9	(12,2 - 13,6)		38,3	(37,2 - 39,4)		57,9	(56,7 - 59,1)	
	Femenino	38.643	21,6	(20,9 - 22,4)		60,6	(59,6 - 61,6)		66,6	(65,7 - 67,6)	
Edad agrupada (años)					0,000			0,000			0,000
	18-29	21.980	9,9	(9,2 - 20,6)		28,2	(27,1 - 29,2)		37,6	(36,6 - 38,7)	
	30-39	16.230	18,4	(17,3 - 19,5)		51,3	(49,8 - 52,9)		64,9	(63,5 -66,4)	
	40-49	14.573	21,5	(20,3 - 22,9)		61,7	(60,3 - 63,1)		76,1	(74,7 - 77,5)	
	50-59	12.765	23,3	(22,1 - 24,5)		67,7	(66,3 - 69,1)		81,7	(80,4 - 82,8)	
	60-64	4.767	25,2	(22,9 - 27,6)		73,8	(71,3 - 76,1)		85,9	(84,1 - 87,6)	
Cuartil de riqueza					0,000			0,000			0,000
	Más bajo	31.443	16	(15,4 - 16,7)		47,6	(46,4 - 48,8)		61,6	(60,5 - 62,6)	
	Bajo	16.618	18,2	(17,3 - 19,2)		49,2	(47,9 - 50,5)		61,5	(60,1 - 62,8)	
	Medio	13.406	19,1	(18,0 - 20,3)		53,3	(51,7 - 54,9)		65,3	(63,8 - 66,7)	
	Alto	8.848	17,2	(15,9 - 18,7)		51,2	(49,2 - 53,3)		62,1	(59,8 - 64,4)	
Etnia					0,000			0,000			0,000
	Negro-Afro ++	6.420	21,6	(20,2 - 23,1)		50,8	(48,7 - 52,8)		58,5	(56,3 - 60,7)	
	Indígena	5.460	14,5	(12,3 - 17)		45,9	(41,3 - 50,6)		66,2	(63,6 - 68,6)	
	Sin pertenencia	57.809	17,3	(16,7 - 17,9)		50,3	(49,4 - 51,2)		62,8	(61,9 - 63,7)	
Área residencia					0,000			0,000			0,546
	Urbano	52.899	18,2	(17,5 - 18,9)		50,9	(50 - 51,9)		62,7	(61,7 - 63,6)	
	Rural	17.416	15,2	(14,4 - 16,1)		47,4	(45,9 - 49)		62,2	(60,9 - 63,5)	
Región					0,000			0,001			0,023
	Atlántica	19.505	18,8	(18 - 19,7)		53,2	(51,9 - 54,5)		63,4	(62,3 - 64,5)	
	Oriental	10.314	16,2	(15 - 17,6)		51,9	(49,5 - 54,3)		64,9	(62,6 - 67,3)	
	Orinoquia	9.966	20,8	(19,1 - 22,6)		50	(47,7 - 52,3)		63,6	(61,2 - 66)	
	Bogotá	4.388	15,6	(13,6 - 17,9)		49,2	(46,5 - 52)		63	(60 - 65,8)	
	Central	16.407	16,7	(15,8 - 17,6)		48,7	(47,4 - 49,9)		60,5	(59,4 - 61,6)	
	Pacífica	9.735	20	(18,7 - 21,3)		47,8	(45,8 - 49,7)		61,6	(59,6 - 63,5)	

IMC: índice de masa corporal circunferencia de cintura; CC: circunferencia de cintura; ICE: índice cintura-estatura

+ Número de individuos sin ponderar. Los porcentajes tienen en cuenta la ponderación y las etapas de diseño.

++ Integra: negro, afrocolombiano, afrodescendiente, mulato y palenquero de San Basilio

^*^ Valores de p, comparaciones realizadas con la prueba de ji al cuadrado (*x* ^2^)

La misma tendencia se observó en los resultados por variables de desagregación: se encontró que todas las clasificaciones de obesidad abdominal según el índice cintura-estatura eran mayores al compararlas con las obtenidas según la circunferencia de la cintura. También, se resalta que las prevalencias más grandes para todos los indicadores se encontraron en las mujeres, en los individuos de mayor edad (60 a 64 años), para el cuartil de riqueza medio y para el área de residencia urbana.

Al diferenciar según el tipo de obesidad, además de las prevalencias más grandes mencionadas, se destacan: en la general (IMC), la obesidad en los afrodescendientes (21,6 %) y en la región de la Orinoquía y la Amazonía (20,8 %); en la obesidad abdominal según la circunferencia de la cintura, también resaltan la prevalencia según la etnia en los afrodescendientes (50,8 %) y en la región Atlántica (53,2 %); en la obesidad abdominal según el índice cintura- estatura, la etnia indígena (66,2 %) y la región Oriental (64,9 %) presentaron las mayores proporciones (cuadro 3).

Según la edad, las prevalencias más bajas son para los más jóvenes (18 a 29 años) y van aumentando con la edad. Para la desagregación por región, entre las tres clasificaciones de obesidad no se observa un comportamiento similar: la menor prevalencia para la obesidad general se dio en la región Atlántica (18,8 %), mientras que, para la obesidad por circunferencia de la cintura estuvo en la región del Pacifico (47,8 %) y, según el índice cintura- estatura, en la región central (60,5 %).

## Discusión

En la comparación de las medidas e índices antropométricos evaluados, se encontró que las prevalencias de obesidad son mayores con la circunferencia de la cintura y el índice cintura-estatura, y que hay relaciones significativas con las variables de análisis. Se resalta, por ejemplo, que a medida que aumenta la edad, también lo hace la prevalencia de obesidad; las cifras son superiores en mujeres, en el área urbana y en el cuartil medio de riqueza.

Las principales medidas e índices antropométricos mostraron algunos aspectos que vale la pena resaltar. Aun cuando en los hombres el peso y la estatura son mayores, el IMC y el índice cintura-estatura lo son en las mujeres. Además, los promedios fueron superiores a los puntos de corte utilizados. Esto sugiere que, en la población adulta, tanto hombres como mujeres, el valor calculado del riesgo es igual o superior a 0,5, lo que denota que existen factores de riesgo para enfermedad cardiovascular, como afirman Hernández y Huerta para el caso del índice cintura-estatura ^13^. No obstante, las medias del IMC y del índice cintura-estatura, son inferiores a las reportadas en otros estudios que presentan valores superiores a 26,8 kg/m^2^ y 0,6, respectivamente ^7^^,^^9^.

Se observó que los individuos clasificados con obesidad general tienen medias superiores en las medidas de peso, IMC, circunferencia de cintura e índice cintura-estatura, que los clasificados con obesidad abdominal. Esto podría estar asociado a que el IMC no solo tiene en cuenta la masa grasa localizada, sino que se ve influenciado por el componente muscular, el cual puede variar entre los individuos según su edad, sexo y actividad física. Además, se ha encontrado que, en algunas situaciones, con el IMC se sobreestima la obesidad en individuos no caucásicos y, consecuentemente, se altera su asociación como factor de riesgo ^10^.

Los resultados de obesidad general de este estudio fueron similares a los de la Encuesta Europea de Salud para España, en la cual se reportó una prevalencia del 16,9 % ^16^; no obstante, fueron inferiores a los reportados por otros países, como Chile en su encuesta de salud que fue del 31,2 % ^17^ y México en la Encuesta de Salud y Nutrición que fue del 36,1 % ^18^. Estas diferencias pueden estar influenciadas por factores ambientales, económicos y de estilos de vida, que afectan la dinámica en la que se presenta la obesidad.

Según nuestros resultados, aproximadamente la mitad de los adultos en Colombia tienen obesidad abdominal. Esto se debe, en parte, a un importante consumo de alimentos con gran contenido calórico, que es la causa más frecuente de obesidad central o visceral, ya que la energía excesiva se almacena en los adipocitos. Así se reporta en un estudio en Venezuela, en el cual se utilizaron puntos de corte similares y se obtuvo una obesidad abdominal superior (86 %), así como una correlación fuerte con el índice cintura-estatura y el IMC; además, mencionan que por cada 5 cm que se aumenta la circunferencia de cintura, el riesgo de muerte se incrementa en 13 % para las mujeres y en 17 % para los hombres ^19^.

Por otra parte, en una población adulta del sur de México, la prevalencia de obesidad abdominal según la circunferencia de cintura (61,8 %) fue superior a la obtenida en el presente estudio, aun cuando sus puntos de corte fueron superiores a los utilizados en Colombia. Sin embargo, se encontró que los individuos con obesidad abdominal pueden tener dos veces más riesgo de presentar un síndrome metabólico ^10^.

En cuanto a la obesidad abdominal según el índice cintura-estatura, es 11,4 puntos porcentuales mayor que la encontrada con la circunferencia de cintura. Este hallazgo puede estar asociado con la incorporación de la estatura en el índice cintura-estatura, lo que permitiría diagnosticar mejor el riesgo asociado con la obesidad abdominal, y valorar la distribución y acumulación de la grasa corporal ^7^. La proporción de obesidad abdominal en el presente estudio, coincide con lo reportado en otras publicaciones ^1^^,^^2^^,^^19^, en las cuales los porcentajes son superiores al 60 %. Además, se comporta como en el estudio de Martínez *et al*., el cual mostró que un índice cintura-estatura elevado se relaciona con un mayor riesgo cardiometabólico (hipertriacilgliceridemia, OR=2,9; HDL baja, OR=2,3), y con el sobrepeso y la obesidad (OR=16) ^20^. De igual manera, se encontró que un índice cintura-estatura elevado indicaba ocho veces más riesgo de un problema cardiovascular y tres veces más riesgo de una alteración aterogénica.

Llama la atención que, en la población colombiana, mientras una quinta parte presentaba obesidad general, uno de cada dos adultos presentó obesidad abdominal. Estos resultados cobran importancia por la evidencia que ha demostrado que la obesidad central o abdominal es una característica común ^2^ y la expresión más frecuente de insulino-resistencia; además, es un factor etiopatogénico de diabetes mellitus ^1^ y, también, de complicaciones metabólicas, lo cual favorece la presencia de hipertensión arterial sistémica y factores de riesgo para enfermedad cardiovascular ^2^^,^^21^.

Respecto a otras variables, se encontró que, a medida que aumenta la edad, se incrementa la prevalencia de obesidad; esto se puede explicar porque, al avanzar la edad, el gasto calórico disminuye, por lo cual se tiende a acumular mayor cantidad de masa grasa, principalmente como depósito visceral ^22^. Estos hallazgos se asocian con cambios característicos en el envejecimiento, también referidos en un estudio en Ecuador ^23^ y otro en Trujillo (Perú); además, se encontró que el riesgo de síndrome metabólico se incrementa con la mayor edad ^1^. Según el “Análisis de situación de salud para Colombia”, el envejecimiento refleja la exposición prolongada a factores de riesgo que generan un incremento en la morbimortalidad por enfermedades no trasmisibles y en la atención en salud que estas requieren ^24^.

En este estudio, ser mujer es otro factor asociado tanto con la obesidad general como con la abdominal; este resultado coincide con otros reportes sobre población adulta, en los cuales el porcentaje para obesidad abdominal fue mayor en las mujeres (25-27). Las diferencias entre hombres y mujeres pueden estar relacionadas con características biológicas, psicológicas, metabólicas y hormonales, que las hacen más propensas a presentar obesidad ^28^. Además, como se encontró en la ENSIN 2015, son las mujeres las que presentan el menor cumplimiento de la recomendación de actividad física (16,4 %), en comparación con los hombres (29,5 %) ^4^.

Otro factor sociodemográfico asociado con mayor prevalencia de obesidad es la zona geográfica donde se encuentra la población estudiada, ya que los ubicados en el área urbana presentaron las mayores prevalencias, lo cual concuerda con los resultados de un estudio en Perú ^8^, el cual mostró que la obesidad general (22,5 %) y la abdominal (37,4 %) son superiores a las observadas en personas que viven en el área urbana. Esta situación puede explicarse por un mayor acceso a alimentos procesados, mayor tiempo fuera de la casa y, por lo tanto, un alto consumo de alimentos fuera del hogar; a esto se suma un mayor tiempo frente a pantallas, del 61,7 % en el área rural, en comparación con el 40,2 % en el área urbana ^4^.

Además de los factores mencionados anteriormente, características como la etnia también influyen en la distribución de grasa corporal a nivel general ^29^. En la presente investigación, los que se autorreconocieron como afrodescendientes presentaron mayor obesidad general y abdominal según la circunferencia de cintura, y los indígenas, mayor prevalencia de obesidad abdominal según el índice cintura-estatura.

Teniendo en cuenta que con el índice cintura-estatura se encontraron las mayores prevalencias de obesidad abdominal, en comparación con otros métodos como la circunferencia de cintura, la que no permite diagnosticar a toda la población que puede presentar riesgo cardiovascular. Con los hallazgos del presente estudio y lo referido en la literatura ^9^^,^^23^^,^^19^^,^^20^, se puede plantear que, con el índice cintura-estatura se identifica un mayor número de personas clasificadas con obesidad abdominal, es decir, es un mejor indicador del riesgo, lo que permite intervenirlas de forma oportuna.

En este estudio, utilizando el índice cintura-estatura, se presentaron mayores porcentajes de obesidad abdominal en mujeres, al igual que en las personas mayores, lo que permite el análisis diferenciado por sexo y grupos de edad.

En conclusión, la obesidad abdominal, independientemente del indicador utilizado, es cerca de tres veces superior a la obesidad general por IMC y el índice cintura-estatura identifica más personas, en especial hombres con obesidad abdominal, en comparación con la circunferencia de cintura.

Una limitación del estudio es el diseño transversal de la ENSIN que, aunque no afecta las comparaciones realizadas, impide las inferencias causales entre las variables estudiadas, por lo que los resultados son de naturaleza asociativa y no causal. Es recomendable hacer análisis longitudinales que incluyan también información sobre variables bioquímicas y tensión arterial, así como desarrollar estudios predictivos del punto de corte adecuado con el índice cintura-estatura para la población colombiana. También, es importante tener en cuenta que, a pesar de todos los controles implementados y la estandarización en las mediciones en las encuestas, es posible que se presenten sesgos de selección o errores de medición.

Con respecto a las medidas, la circunferencia de cintura, aunque es muy utilizada para establecer obesidad abdominal, es cuestionada por las diferencias que puede presentar en los puntos de corte, según la población de estudio ^7^.

Es importante analizar de manera diferenciada variables como la edad y el sexo, que pueden influir como factores de confusión en las relaciones entre las medidas antropométricas y los factores de riesgo cardiovascular.

Por otro lado, el encontrar que más de la mitad de la población analizada tuvo obesidad abdominal, significa que esa misma proporción está en riesgo cardiovascular.

Estos resultados deben tenerse en cuenta para implementar intervenciones adecuadas que sean focalizadas y específicas para la población colombiana, con el fin de contribuir con mayor impacto a prevenir las enfermedades crónicas y cardiovasculares. En Colombia, estas se consideran la primera causa de carga de enfermedad y muerte, la cual produce un mayor efecto negativo sobre la calidad de vida de estos adultos y genera altos costos para el sistema de salud.
